# Establishing a standard method for analysing case detection delay in leprosy using a Bayesian modelling approach

**DOI:** 10.1186/s40249-023-01065-4

**Published:** 2023-02-20

**Authors:** Thomas Hambridge, Luc E. Coffeng, Sake J. de Vlas, Jan Hendrik Richardus

**Affiliations:** Department of Public Health, Erasmus MC, University Medical Center Rotterdam, Rotterdam, The Netherlands

**Keywords:** Leprosy, Case detection delay, Neglected tropical diseases, Epidemiological methods, Bayesian approach, Statistical model

## Abstract

**Background:**

Leprosy is an infectious disease caused by *Mycobacterium leprae* and remains a source of preventable disability if left undetected. Case detection delay is an important epidemiological indicator for progress in interrupting transmission and preventing disability in a community. However, no standard method exists to effectively analyse and interpret this type of data. In this study, we aim to evaluate the characteristics of leprosy case detection delay data and select an appropriate model for the variability of detection delays based on the best fitting distribution type.

**Methods:**

Two sets of leprosy case detection delay data were evaluated: a cohort of 181 patients from the post exposure prophylaxis for leprosy (PEP4LEP) study in high endemic districts of Ethiopia, Mozambique, and Tanzania; and self-reported delays from 87 individuals in 8 low endemic countries collected as part of a systematic literature review. Bayesian models were fit to each dataset to assess which probability distribution (log-normal, gamma or Weibull) best describes variation in observed case detection delays using leave-one-out cross-validation, and to estimate the effects of individual factors.

**Results:**

For both datasets, detection delays were best described with a log-normal distribution combined with covariates age, sex and leprosy subtype [expected log predictive density (ELPD) for the joint model: −1123.9]. Patients with multibacillary (MB) leprosy experienced longer delays compared to paucibacillary (PB) leprosy, with a relative difference of 1.57 [95% Bayesian credible interval (BCI): 1.14–2.15]. Those in the PEP4LEP cohort had 1.51 (95% BCI: 1.08–2.13) times longer case detection delay compared to the self-reported patient delays in the systematic review.

**Conclusions:**

The log-normal model presented here could be used to compare leprosy case detection delay datasets, including PEP4LEP where the primary outcome measure is reduction in case detection delay. We recommend the application of this modelling approach to test different probability distributions and covariate effects in studies with similar outcomes in the field of leprosy and other skin-NTDs.

**Graphical Abstract:**

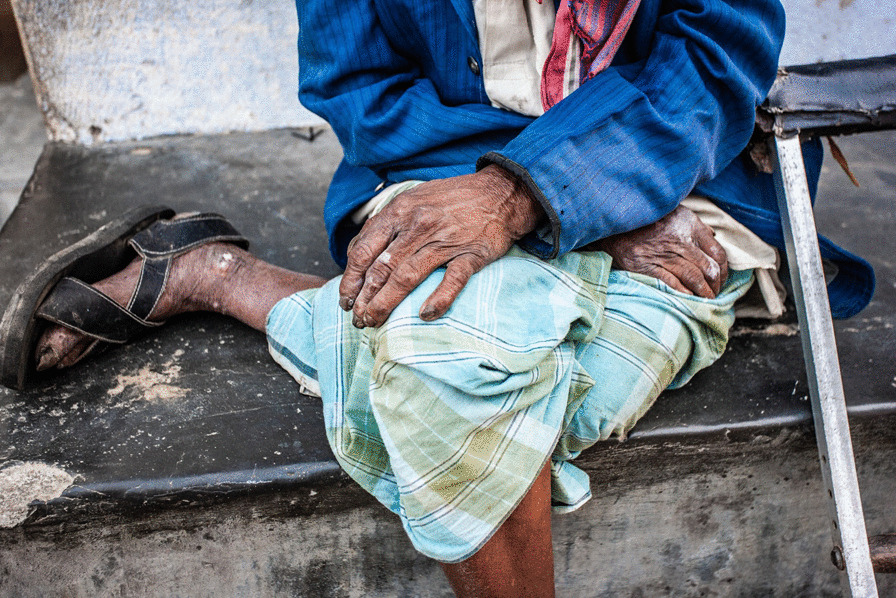

**Supplementary Information:**

The online version contains supplementary material available at 10.1186/s40249-023-01065-4.

## Background

Leprosy is an infectious disease caused by *Mycobacterium leprae* that mainly affects the skin and peripheral nerves [1]. The disease remains a source of preventable disability in many endemic countries and is formally recognised by the World Health Organization (WHO) as a neglected tropical disease (NTD) [2]. At the individual level, delay in diagnosis and treatment of leprosy often results in negative outcomes, such as physical impairment and disability [3]. Leprosy has a very long incubation period due to the slow growth of *M. leprae*, which can range from 1 to more than 20 years [4]. Case detection delay in leprosy is defined as the total time from first signs of leprosy until diagnosis. Together, case detection delay is comprised of two components: the patient delay (the period between noticing the first sign or symptom to the first health care provider consultation) and the health system delay (the period between first health care provider consultation and the final diagnosis of leprosy) [5]. Assessing an individual’s case detection delay is becoming a more widely used indicator in leprosy research as shortening this interval is imperative to interrupting transmission and preventing disability [1]. In fact, case detection delay will be used as the main outcome measure of effectiveness in the post exposure prophylaxis for leprosy (PEP4LEP) study, in which two integrated skin screening interventions will be compared in Ethiopia, Mozambique and Tanzania [6]. Unlike tuberculosis, where case detection delay is typically measured in days, delay in leprosy diagnosis is usually measured in months considering the long incubation period of *M. leprae*, the gradual onset of first signs and symptoms, as well as ongoing stigma in many populations that can present barriers to seeking healthcare [5, 7–9].

There are a number of factors that may contribute to delayed case detection in leprosy. These include socioeconomic and community factors, health system capacity and individual factors such as age, sex, clinical subtype and disability [10]. The diagnosis of leprosy is based on clinical signs according to WHO classification criteria, with paucibacillary (PB) defined as five or less skin patches with loss of sensation and multibacillary (MB) defined as six or more skin patches with loss of sensation and/or an affected nerve [11]. It has been previously shown that individuals presenting with MB leprosy have longer detection delays on average and are more likely to present with visible disability, such as nerve impairment and sensory loss, compared to those with the less severe PB form of the disease [12, 13]. A number of studies have reported patient age as a factor associated with delayed case detection, particularly those aged 50 years and above [12, 14, 15]. A person’s sex has also been suggested to be associated with longer delays. While some studies have reported significantly higher rates of grade 2 disability (G2D) in males compared to females, these figures are expected to vary between contexts and are linked to cultural and societal factors, as well as healthcare seeking behaviour [16–18].

Despite recent efforts to develop tools to more accurately estimate case detection delays, no standard method exists to effectively analyse delay data and to compare these between settings and interventions [19]. Statistical modelling has been a useful tool in addressing analogous problems in infectious disease research, including analysing determinants of patient diagnosis delay in tuberculosis, quantifying effects on onset-to-diagnosis waiting times in visceral leishmaniasis and identifying associations between diagnosis delay and neurological outcomes in cryptococcosis [20–22]. In particular, Bayesian statistical modelling is becoming more widely used as an alternative to frequentist statistics, including delayed reporting of count data [23]. Another recent example was a study on dengue fever incidence in Brazil that used a Bayesian hierarchical modelling approach to correct reporting delays and quantify uncertainty [24]. In the field of leprosy, a Bayesian framework has also been applied to predict future sub-clinical and clinical infections in the context of Thailand using a back-calculation model with assumptions for the distributions of incubation period and detection delay [25]. Among the advantages of this approach is the ability to incorporate prior knowledge about parameters into the model and the explicit use of probability to model uncertainty [26, 27].

In this study, we aim to evaluate the characteristics of two existing case detection delay datasets collected from across different contexts. Bayesian models were fit to each dataset to assess which probability distribution best describes variation in observed case detection delays and to estimate the effects of individual factors. This approach could be used as a standard methodology to compare the effectiveness of the two interventions in the PEP4LEP study and applied to future research projects with similar outcomes in the field of leprosy and other skin-NTDs.

## Methods

### Datasets

Two sets of leprosy case detection delay data that were recently collected and available to the researchers were used to evaluate characteristics. The first was collected from the baseline component of the PEP4LEP study, a cluster-randomised implementation trial comparing two interventions of integrated skin screening combined with single-dose rifampicin post-exposure prophylaxis (SDR-PEP) for contacts of leprosy patients in Ethiopia, Mozambique, and Tanzania [6]. This dataset (‘PEP4LEP dataset’) was comprised of 181 estimates of case detection delay in patients diagnosed between 2020 and 2021 living in highly endemic areas obtained from interviews with a structured questionnaire within six months of diagnosis [19, 28–30]. The second dataset contained self-reported delays in the health facility at the time of diagnosis from 87 individuals presented in a recent systematic review investigating case characteristics during the declining stages of leprosy incidence, with diagnosis dates ranging from 1989 to 2018 [31]. The case reports in this dataset (‘Global dataset’) were collected from 8 low endemic countries: Australia, Germany, Italy, Japan, Portugal, Spain, United Kingdom and United States.

All estimates were presented in months and both included data marked as 0 for no delay, as well as decimals for delays reported in weeks. Left censoring was applied to values of less than or equal to one month, since a detection delay of exactly zero cannot exist in reality and these values must reflect a delay that is shorter than the minimal non-zero delay. This is because there must be some inherent delay that may be less than one month but more than no time at all until diagnosis, namely the time elapsed before visiting a health facility. Additionally, the reason for censoring non-null values less than or equal to one month rather than one, two or three weeks is that the majority of leprosy case detection delay estimates are reported in months, including those from the structured questionnaire, so we are censoring any delays below the lowest unit value. No right censoring was applied to values above a certain range that may be considered statistical outliers, since very long delays are expected in the case of leprosy. Such ‘lifelong delays’ may be true estimates reported by individuals that have experienced clinical manifestations of leprosy for multiple years or decades prior to diagnosis.

### Statistical analyses

First, the characteristics of both datasets were examined, including their distribution, variance and measures of central tendency, primarily using histograms of case detection delay data. Additionally, the cumulative distribution function (CDF) of case detection delay and log-transformed case detection delay were examined to further evaluate the distribution of both data sets. Based on these observations, three positively bounded, continuous probability distributions for the response variable were fitted as statistical models to each case detection delay dataset: a log-normal distribution (i.e., a normal distribution on the logarithmic scale), a gamma distribution and a Weibull distribution [32]. We included age (in decades), sex and leprosy subtype (PB vs MB) as predictors in all three models since these variables are widely available for leprosy cases and have been reported to be associated with the length of case detection delay [10]. Leprosy subtype was assigned in both datasets according to the WHO classification system [11]. In each of the models, age, sex and leprosy subtype were used as linear predictors of the log-mean, meaning that the exponent of any model coefficient reflected the relative change in mean detection delay (geometric mean for log-normal model and arithmetic mean for the two other distributions) for each unit change in the value of a predictor, keeping the other predictors fixed. The assumption of a linear relationship between age and log-detection delay was assessed using a scatterplot with line of best fit. Left censoring of case detection delays less than or equal to one month was explicitly modelled. Left-censored observations were modelled by defining their likelihood as the cumulative probability of the detection delay being between zero and 1 month, given the expected detection delay for that observation based on the predictors and the standard deviation of unexplained variation in detection delays in the entire dataset (which was mostly informed by the non-censored observations). Model parameters were estimated in a fully Bayesian framework in R version 4.1.3 (https://www.r-project.org/) [33], using the package brms version 2.17.0 [34]. The brms package utilises the Hamiltonian Monte Carlo sampling for exploring the posterior via the No-U-Turn Sampler [35].

To check that prior distributions were reasonable, prior predictive checks were performed to check whether the specification of the priors for the individual model parameters also make sense in terms of the resulting expected prior distribution of data (i.e., our prior expectation of what a typical dataset might look like). Ideally, the resulting distribution would have most of its probability mass span a plausible range of datasets. [36]. The following combinations were tested as priors for the model intercept and regression coefficients to check if the generated predicted values spanned a plausible range that fit with our prior expectations: normal distribution [mean = 0, standard deviation (*SD*) = 1], normal distribution (mean = 0, *SD* = 5) and normal distribution (mean = 0, *SD* = 10). In addition, we performed a series of posterior predictive checks (PPCs) to assess whether the models were capable of producing valid predictions [36]. In the PPC procedure, for each posterior draw of model parameters, we generated a synthetic dataset of the same dimensions as the original, drawing from the fitted probability distribution, conditional on observed distribution of predictors in the original data. The model fits were then be assessed by comparing (graphical) summaries of the repeated synthetic datasets with the same type of summary of the original data. For these comparisons, we plotted the empirical cumulative distributions, kernel density estimates, histograms of means and medians, and scatterplots of means versus standard deviations of observed and predicted detection delays. Pareto smoothed importance sampling (PSIS) was used to assess the reliability of model estimates by highlighting data points that were most influential on the posterior distribution, with an acceptable Pareto shape k threshold of 0.5 used for the diagnostic output of the model [37]. Finally, model diagnostics were performed to check model validity, including trace plots for parameters and autocorrelation plots to assess sampling dependency of Markov chains. All models had the following run specifications: number of Markov chains = 4, iterations = 10,000, burn-in (warmup) time = 1000 and all parameters were initialised to zero.

As a quantitative assessment of the external validity of each model, we then assessed the pointwise out-of-sample prediction accuracy based on approximate leave-one-out cross-validation (LOO-CV). This method uses the expected log predictive density (ELPD), related to the posterior predictive density, to calculate the LOO information criterion (LOOIC) for new data, with corresponding standard errors (SE) as a measure of uncertainty for the criterion. The LOOIC is equal to − 2 × ELPD and a lower value of the LOOIC means that a model explains the variation in the data better. Based on the SEs of the difference between two models’ LOOIC, the performance of those models can be compared, while penalising for model complexity (overfitting inflates the SE of the ELPD) [38]. In case of conflicting results with regard to selection of the distribution for the two datasets, the fit to the PEP4LEP dataset took precedence over the Global dataset, given that these delays were obtained through interviews with a structured questionnaire and were therefore deemed more reliable. Once the final model was selected based on validity checks for both datasets separately, the relative difference between the two datasets (PEP4LEP and Global) was estimated by merging the two datasets and adding ‘dataset’ as a binary indicator covariate in the model (in addition to age, sex and leprosy subtype). To account for the different residual variation observed in each dataset, we specified in our model that the residual standard deviation (sigma) parameter of the response distribution could vary by dataset. We also generated conditional effect plots, which allow us to plot the predicted geometric means of the response conditional on all other predictors. The conditional values used to fix the other predictors were the arithmetic mean of age (decades) for the joint dataset and the reference category for the sex variable (male).

## Results

### Characteristics of leprosy case detection delay data

An overview of case detection delay data and patient age for both datasets, including measures of central tendency and variability can be seen in Table 1. While the arithmetic mean detection delay was higher in the Global dataset, the geometric mean and the median delay were lower compared to the PEP4LEP dataset. The coefficient of variation (CV) was also higher in the Global dataset. This difference was primarily driven by a few cases with very long delays in the Global dataset, including one patient with a reported delay of 396 months (33 years). Leprosy cases in the Global dataset were slightly older, with an average age of 47.6 years compared to 39.7 years in the PEP4LEP dataset. In Table 2, a summary of case characteristics and detection delays across different age groups, sex and leprosy subtype is shown, including the arithmetic mean, geometric mean and median for each subgroup. In both datasets, around two-thirds of leprosy cases were male. A majority of all cases were MB, with a higher proportion observed in the PEP4LEP dataset compared to the Global dataset (84.5% and 75.3% respectively).

**Table 1:** Overview of case detection delay and age for individuals in the PEP4LEP and Global datasets

Case detection delay (months)											Age (years)	
Data source	*N*	Arithmetic mean	Geometric mean^a^	Standard deviation	Coefficient of variation	95% Confidence intervals (arithmetic mean)		Median	Range			
						Lower	Upper		Min	Max	Arithmetic mean	Median
PEP4LEP dataset	181	25.9	18.2	28.7	1.11	21.7	30.0	18.0	0	292	39.7	36.0
Global dataset	87	30.6	13.9	54.8	1.79	19.0	42.1	14.0	0	396	47.6	42.0

^a^Geometric mean of non-zero values (number of zero values in datasets: PEP4LEP = 1, Global = 2). *PEP4LEP* Post exposure prophylaxis for leprosy

**Table 2:** Overview of case characteristics and detection delays across different age groups, sex and leprosy subtype for individuals in the PEP4LEP and Global datasets

Characteristic	Data source							
	PEP4LEP				Global			
	*N* (%)	Case detection delay (months)			*N* (%)	Case detection delay (months)		
		Arithmetic mean	Geometric mean^a^	Median		Arithmetic mean	Geometric mean^a^	Median
Age group								
0‒20	20 (11.0)	22.3	16.6	17.5	3 (3.4)	24.7	17.9	20.0
21‒40	91 (50.3)	22.8	17.7	18.0	37 (42.5)	21.6	11.8	12.0
41‒60	48 (26.5)	30.7	17.1	18.0	19 (21.8)	35.2	21.4	24.0
> 60	22 (12.2)	31.4	24.9	27.5	28 (32.2)	39.9	12.4	12.0
Sex								
Male	116 (64.1)	24.7	17.8	18.0	60 (69.8)	29.7	13.1	12.5
Female	65 (35.9)	28.0	18.9	18.0	26 (30.2)	32.8	16.0	24.0
Leprosy subtype								
PB	28 (15.5)	18.9	13.7	12.5	21 (24.7)	16.0	8.6	17.0
MB	153 (84.5)	27.1	19.1	20.0	64 (75.3)	35.3	16.1	13.5

*PB* Paucibacillary, *MB* Multibacillary, *PEP4LEP* post exposure prophylaxis for leprosy

^a^Geometric mean of non-zero values (number of zero values in datasets: PEP4LEP = 1, Global = 2). *MB* Multibacillary, *PB* Paucibacillary, *PEP4LEP* Post exposure prophylaxis for leprosy

**Fig. 1:**
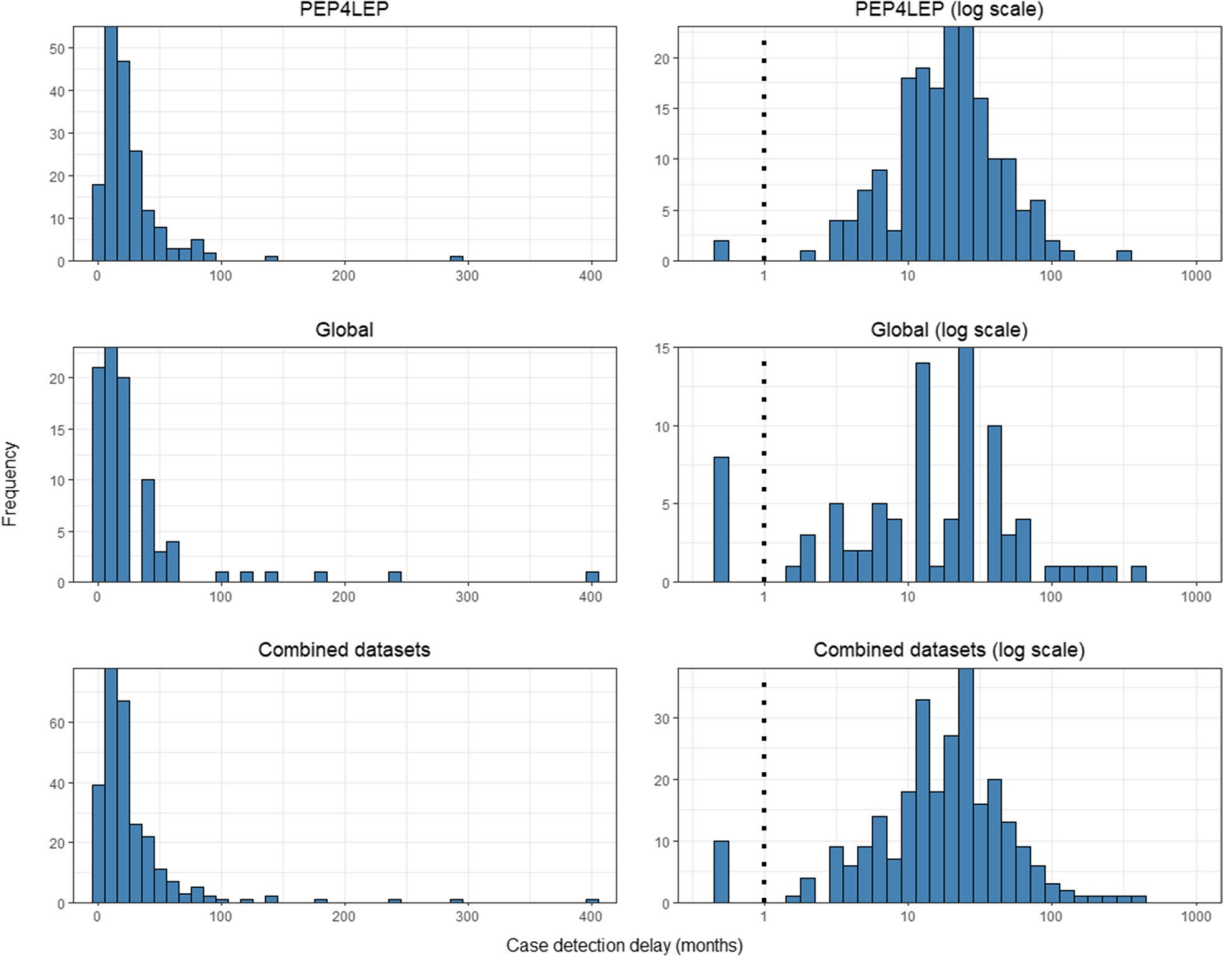
Distributions of case detection delay (months) for the PEP4LEP, Global and combined datasets. The data are presented on the natural scale (left) and the logarithmic scale (right). The blacked dashed line indicates the point of left-censoring, with these values plotted at half of the censored value (0.5)

Histograms showing the distributions of case detection delay for both datasets, as well as the combined datasets, are presented above (Fig. 1). In both datasets, detection delays were non-normally distributed with a right-skew on the natural scale. For the Global dataset, there was a higher proportion of delays under 24 months and a few very long delays. The PEP4LEP dataset resembled a normal distribution when plotted on the log scale, suggesting a possible log-normal distribution. A similar distribution can be seen for the Global dataset when plotted on the log scale, although the pattern was less clear. The CDF of case detection delay and log-transformed case detection delay were also examined (Additional file 1: Fig. S1). The CDF of case detection delay on the natural scale was indicative of an exponential distribution for both datasets. The CDF of the log-transformed case detection delay showed a sigmoid curve, particularly for the PEP4LEP dataset, suggesting a log-normal distribution.

### Statistical modelling

Before including age in the model, we first checked that the linear association between age and log-outcome was a reasonable assumption (Additional file 1: Fig. S2). For both datasets, as well as the combined datasets, LOO-CV showed that the log-normal model had the highest ELPD and lowest LOOIC (Table 3). These measures indicate that the log-normal model was expected to have better predictive performance than the gamma and Weibull models, although there was a large degree of uncertainty (i.e., standard error of the difference). Based on the LOO-CV and PPCs, the log-normal distribution was selected to be used in the joint model for the integrated detection delay datasets.

**Table 3:** Summary of model comparison using leave-one-out (LOO) cross-validation

PEP4LEP dataset				
Model	LOOIC	ELPD	ELPD difference	Standard error (ELPD difference)
Gamma	1532.4	−766.2	−6.5	8.1
Weibull	1543.9	−771.9	−12.2	9.8
Log-normal	1519.5	−759.8	0	0

Global dataset				
Model	LOOIC	ELPD	ELPD difference	Standard error (ELPD difference)
Gamma	738.1	−369.0	−6.3	5.5
Weibull	732.9	−366.4	−3.7	4.1
Log-normal	725.5	−362.7	0	0

Combined datasets				
Model	LOOIC	ELPD	ELPD difference	Standard error (ELPD difference)
Gamma	2255.4	−1127.7	−3.8	8.9
Weibull	2261.7	−1130.8	−7.0	9.5
Log-normal	2247.7	−1123.9	0	0

LOOIC is LOO information criterion; ELPD is expected log predictive density. LOOIC = −2 × ELPD and ELPD difference is the ELPD value relative to that of the best performing model, i.e., the log-normal model, which has the lowest LOOIC (or highest ELPD). The standard error is a measure of uncertainty for the ELPD difference relative to the log-normal model

*ELPD* Expected log predictive density, *LOOIC* LOO information criterion, *PEP4LEP* Post exposure prophylaxis for leprosy

After selecting the log-normal model as the best, we jointly analysed the two datasets in one model with a predictor indicating the dataset. Based on prior predictive checks, we used a normal distribution (mean = 0, *SD* = 1) for the intercept and regression coefficients as these priors generated prior predictive values spanning a plausible range that fit with our prior expectations of what a dataset could reasonably look like, whereas this was not the case with more vague priors (Additional file 1: Fig. S3). PPCs further showed that the joint log-normal model was able to adequately reproduce the data in terms of posterior predictive distribution (Additional file 1: Fig. S4). A summary of joint model run specifications, including the model equation and priors for the intercept and covariate effects is provided (Additional file 2: Table S1). PSIS revealed that all data points were below the acceptable Pareto shape k threshold (0.5) for the diagnostic output of the model (Additional file 1: Fig. S5). Trace plots indicated convergence towards the posterior distribution (Additional file 1: Fig. S6), while autocorrelation plots showed very low sample dependency for each covariate after only a few lags (Additional file 1: Fig. S7).

**Fig. 2:**
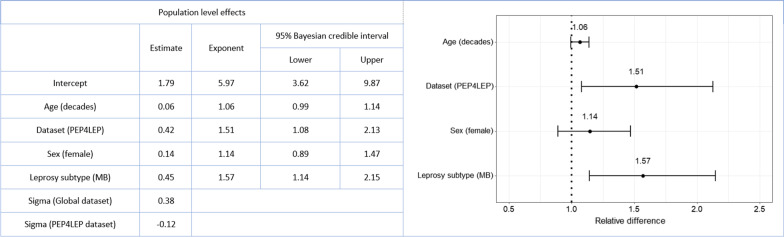
Bayesian estimates of relative difference for covariates in the full model (left) and forest plot of relative differences in geometric mean delays with 95% Bayesian credible intervals for model covariates (right)

**Fig. 3:**
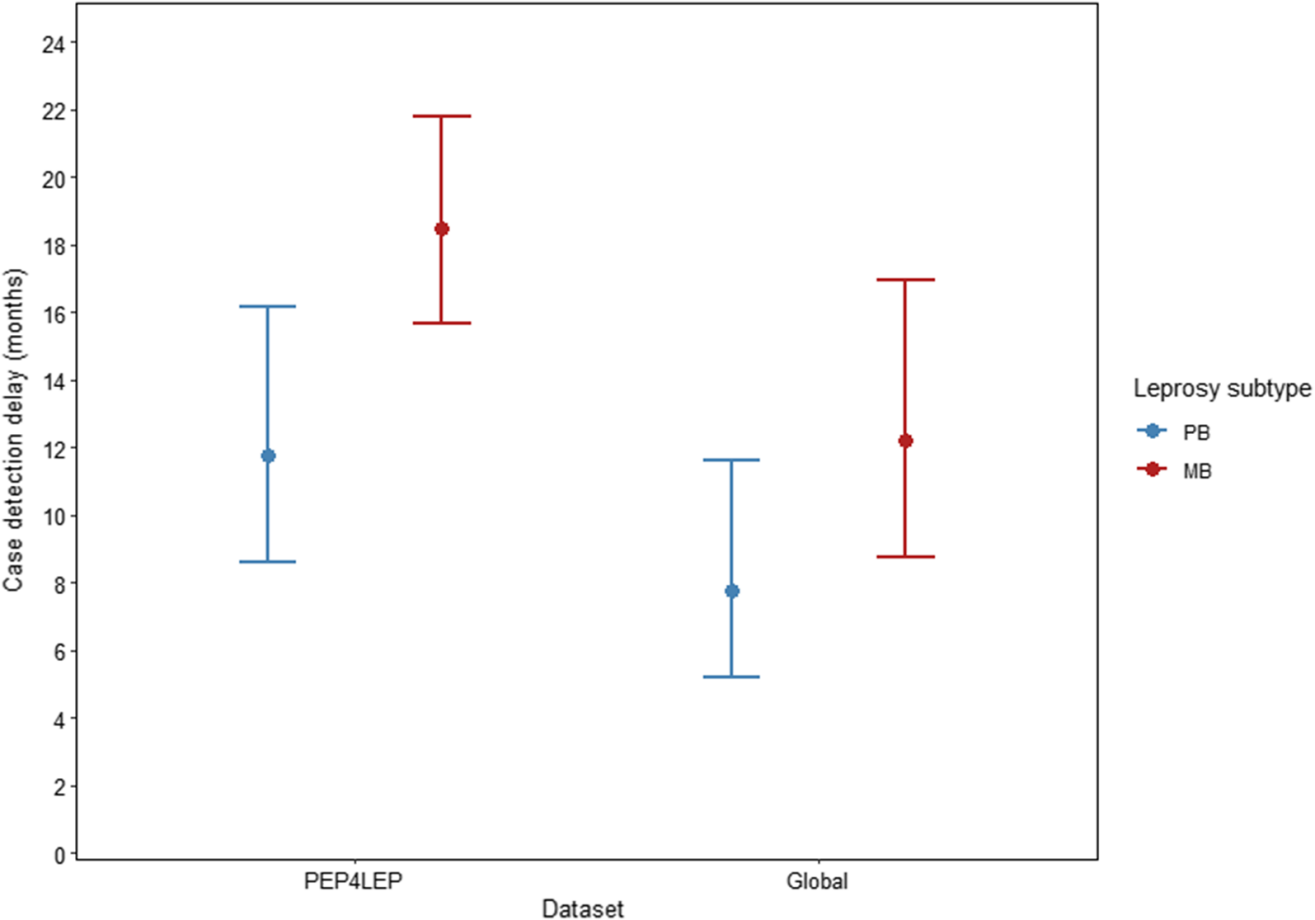
Two-way effect plot for the covariates of the full model: dataset (on the x-axis) and leprosy subtype (PB in blue and MB in red), corrected for age and sex. The solid points represent the geometric mean response values of the posterior predictive distribution with 95% Bayesian credible intervals. *MB* Multibacillary, *PB* Paucibacillary; *PEP4LEP* Post exposure prophylaxis for leprosy

After correction for age, sex, and leprosy subtype, there was a relative difference in the geometric mean case detection delay of 1.51 (95% BCIs: 1.08–2.13) for patients in the PEP4LEP dataset vs the Global dataset (Fig. 2). A longer geometric mean delay was also observed in patients with MB leprosy compared to PB, with a relative difference of 1.57 (95% BCIs: 1.14–2.15). There was no difference between males and females or by age in our model. The joint effects of dataset and leprosy subtype on the geometric mean response values of the posterior predictive distribution, correcting for age and sex, can be seen in Fig. 3. Here we see that the difference in effect (around 1.5 times higher) and level of uncertainty of MB vs PB on case detection delay is more or less the same in the two datasets.

## Discussion

The aim of this study was to evaluate the characteristics of leprosy case detection delay data and select an appropriate model for the variability of detection delays based on the best fitting distribution type. We found that a log-normal distribution best described case detection delay data in the PEP4LEP and Global datasets compared to gamma and Weibull. By fitting a Bayesian model with a log-normal distribution for the response variable, we were able to estimate the relative difference in geometric mean detection delay between the two datasets while correcting for the effects of age, sex and leprosy subtype. This same methodology could be used to compare two or more groups of leprosy case detection delay, with implications for studies such as PEP4LEP where case detection delay is used as the primary outcome measure for comparing effectiveness.

In previous studies, multivariable modelling has been applied to analogous indicators in the field of NTDs [20–22]. A key difference in this study was leprosy case detection delays were reported after diagnosis with no distinction between symptomatic and asymptomatic infection, meaning these data were best handled as a continuous variable and not subject to a time-to-event analysis. In the case of tuberculosis, long delays in diagnosis are associated with greater transmission to contacts [39]. Despite the much longer incubation time, the same reasoning can be applied to leprosy as prolonged exposure with somebody living with the disease in the household or community has been shown to increase the risk of infection [40, 41]. Here we incorporated three individual factors: age, sex and leprosy subtype as predictors in our model since they are widely available in case reports and patient databases. Moreover, these factors have been previously shown to be associated with case detection delays. We considered adding additional variables, in particular disability grade. However, WHO disability classification for leprosy can vary between different settings, including the distinction between grade 1 and grade 2, and this information is not always reported, which was also the case in our study.

When assessing which probability distribution best described variation in observed case detection delays, we deemed it imperative that it demonstrated a strong predictive performance for the observed values in the PEP4LEP dataset, as these delays were estimated using a structured questionnaire and culturally validated in the three PEP4LEP study countries using the conceptual framework of Herdman et al. [19, 42]. Nevertheless, we found that models using a log-normal distribution for case detection delay performed best for both datasets. When modelling case detection delays with a log-normal distribution, it is important to consider that the level of variation may well vary between datasets. Therefore, the standard deviation of the log-normal distribution needs to be allowed to vary by data source or site, as was the case here. After validating our full model for the joint datasets through a series of PPCs, we estimated the effects of individual factors on geometric mean case detection delay. Before including age as a continuous variable in our log-normal model, we first checked that there was a linear relationship between age and log-detection delay, albeit with a weak association. In our full model, we found no relative difference in case detection delay by 10-year increase in age. Although it has been previously reported that patient age is a factor associated with delayed case detection, particularly those aged 50 years and above [12, 14, 15], other studies have found that patient delay was independent of age, including in Brazil, Colombia and China [43–45]. There was also no relative difference observed between males and females in our model. While some studies have reported an association between sex and leprosy case detection delay, these findings likely context specific, particularly with respect to cultural and societal factors, as well as healthcare seeking behaviour and access to health services. On the other hand, we found that the geometric mean case detection delay was around 1.5 times higher for patients in the PEP4LEP dataset compared to the Global dataset and for MB patients compared to PB. Moreover, joint effect plots of the posterior predictive distribution showed that the effect and level of uncertainty of MB vs. PB on case detection delay was more or less the same in the two datasets. This is in line with previous studies that reported MB patients having longer detection delays on average compared to PB and gives more confidence to the validity of our datasets and model fit [12, 13].

A strength of the Bayesian modelling approach used in this study was the ability to test multiple distributions on more than one dataset. Moreover, this method has advantages over performing a simple non-parametric test comparing sample means or medians, as it corrects for covariates thought to have an effect on the outcome. There were also some important limitations, namely the accuracy and precision of the delay estimates. While the PEP4LEP dataset used a more systematic method of estimating case detection delay through a structured questionnaire, there is always a risk of patient recall bias, and this was very likely the case with some patient reported delays collected in the Global dataset. There is also the issue of underreporting, regardless of the data source considered. Recent studies have used statistical modelling to correct underreporting in epidemiological data, including a paper by de Oliveira et al. that used a Bayesian approach to estimate underreporting of leprosy in Brazil [46]. Although correction of underreporting is beyond the scope of this study, it is important to highlight this as a recurrent problem in the study of leprosy rates, especially as it pertains to case detection delay. There are also other factors shown to influence case detection delay, including having a lower disease-symptom perception and lack of knowledge, living in a rural area, performing agricultural labour, unemployment and stigma [10]. Although such factors may undoubtedly play a role in an individual’s case detection delay, these data are less commonly available and we decided to omit them given the aim was for us to establish a standard methodology that can be widely used by researchers in the field. Both datasets were selected for this analysis based on availability and the findings reported here, including the log-normal distribution providing the best fit for the analysed data, may not be valid for other databases of leprosy case detection delay. Moreover, only two other candidate distributions (gamma and Weibull) were considered in this study. Additionally, the two databases explored here come from different regions and, in the case of the Global dataset, have a long time span. Therefore, there may be important differences in other factors influencing case detection delay in leprosy that were not explored here, so the issue of heterogeneity needs to be considered. While we found that case detection delays estimated in months were best described by a log-normal distribution combined with covariates age, sex and leprosy subtype, supported by analysis of two datasets, if case detection delays were reported as another time unit, e.g. in days, weeks or years, this could affect the probability distribution selection. Furthermore, although measuring delays in number of months is more practical given the long incubation time of the disease, it does raise concerns regarding precision, with longer delays subject to high variation that can strongly influence point-estimates. However, we expect that this type of variation is well captured by the use of a log-normal distribution, which accommodates this type of highly skewed variation since geometric means are less affected by these very long delays.

## Conclusions

Case detection delay is an important epidemiological indicator in leprosy research and shortening delay in a population could be seen as a proxy for interrupting transmission and preventing disability in a community. The log-normal model presented here could be used to compare relative differences in the geometric means of leprosy case detection delay datasets, including PEP4LEP where the primary outcome measure is reduction in case detection delay. We recommend the application of this modelling approach as a standard methodology to test different probability distributions and covariate effects in future research projects with similar outcomes in the field of leprosy and other skin-NTDs.

## Supplementary Information

**Additional file 1: Figure S1. **Cumulative distribution function (CDF) of case detection delay (months) values in the Global (red) and PEP4LEP (blue) datasets. ** Figure S2.** Scatter plot of age and log-detection delay with a line of best fit applied to check the linearity assumption for the log-normal model in the PEP4LEP (blue) and Global (red) datasets. **Figure S3.** Prior predictive checks performed using the full model for the joint datasets without observed data. **Figure S4.** Posterior predictive checks performed using the full model for the joint datasets. **Figure S5.** Pareto smoothed importance sampling plots for each data point. **Figure S6.** Density (left) and trace (right) plots for model parameters showing a stable sampling distribution across the four chains and convergence towards the posterior distribution. **Figure S7.** Autocorrelation plots for the model over 25 lags.

**Additional file 2: Table S1.** Details of the joint model run specifications in brms, including model formula and priors used for the intercept and covariate effects.

## Data Availability

The data underlying this article will be shared on reasonable request to the corresponding author.

## Abbreviations

CDF: Cumulative distribution function
ELPD: Expected log predictive density
LOO-CV: Leave-one-out cross-validation
LOOIC: Leave-one-out information criterion
MB: Multibacillary
NTD: Neglected tropical disease
PB: Paucibacillary
PEP4LEP: Post exposure prophylaxis for leprosy
PPC: Posterior predictive checks
PSIS: Pareto smoothed importance sampling
SDR-PEP: Single-dose rifampicin post-exposure prophylaxis
*SD*: Standard deviation
*SE*: Standard error
WHO: World Health Organization
