# Supplementary material for: Establishing a standard method for analysing case detection delay in leprosy using a Bayesian modelling approach
Source: Infect Dis Poverty. 2023 Feb 20;12:12. doi: 10.1186/s40249-023-01065-4 (PMC9940321; doi:10.1186/s40249-023-01065-4)
Supplement: Supplementary file 1 — Additional file 1: Figure S1. Cumulative distribution function (CDF) of case detection delay (months) values in the Global (red) and PEP4LEP (blue) datasets. Figure S2. Scatter plot of age and log-detection delay with a line of best fit applied to check the linearity assumption for the log-normal model in the PEP4LEP (blue) and Global (red) datasets. Figure S3. Prior predictive checks performed using the full model for the joint datasets without observed data. Figure S4. Posterior predictive checks performed using the full model for the joint datasets. Figure S5. Pareto smoothed importance sampling plots for each data point. Figure S6. Density (left) and trace (right) plots for model parameters showing a stable sampling distribution across the four chains and convergence towards the posterior distribution. Figure S7. Autocorrelation plots for the model over 25 lags. [file 40249_2023_1065_MOESM1_ESM.docx]

**Additional file 1**


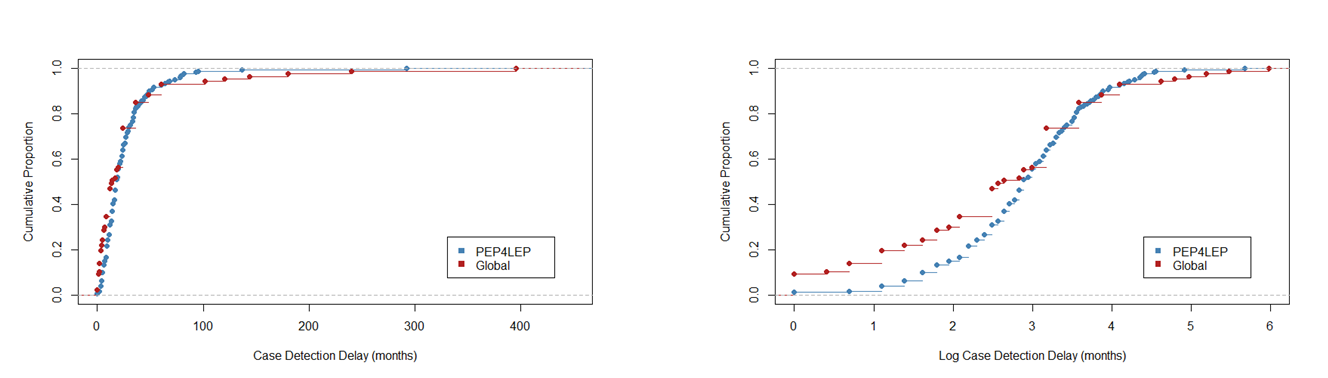


**Figure S1.** Cumulative distribution function (CDF) of case detection delay (months) values in the Global (red) and PEP4LEP (blue) datasets. The CDF plot on the left shows case detection delay presented on the natural scale, while the CDF plot on the right shows the log-transformed case detection delays with left-censored values denoted as zero.


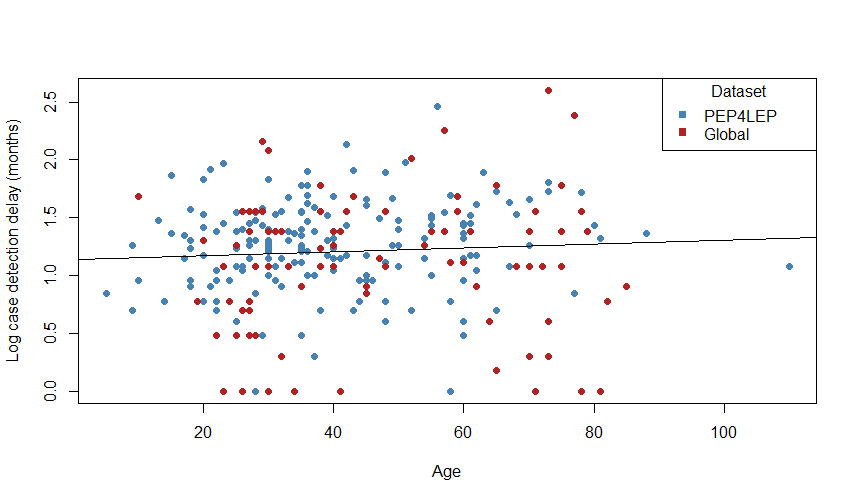


**Figure S2.** Scatter plot of age and log-detection delay with a line of best fit applied to check the linearity assumption for the log-normal model in the PEP4LEP (blue) and Global (red) datasets. The plot shows a weak association between age and log-detection delay for both datasets.

**
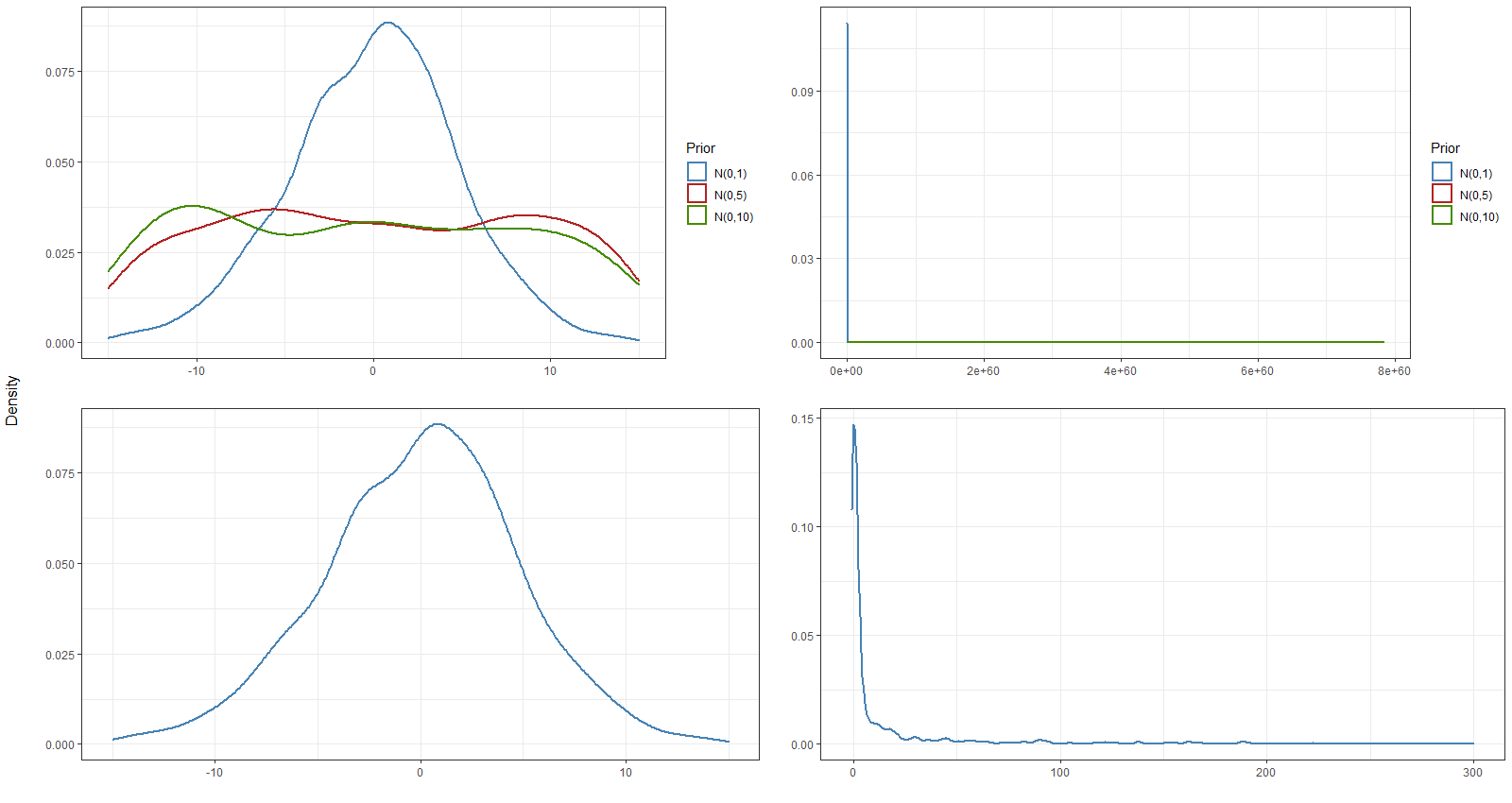
**

**Figure S3.** Prior predictive checks performed using the full model for the joint datasets without observed data. The top left plot shows the kernel density estimates for the intercept of 1000 simulated datasets for three combinations of priors for the model intercept and regression coefficients: normal distribution (mean = 0, SD = 1) in blue, normal distribution (mean = 0, SD = 5) in red and normal distribution (mean = 0, SD = 10) in green, while the top right plot shows the exponents of these same estimates. The bottom two plots shows the same estimates for only normal distribution (mean = 0, SD = 1) priors for the model intercept and regression coefficients, demonstrating that these predicted values span a plausible range that fits with our prior expectations.


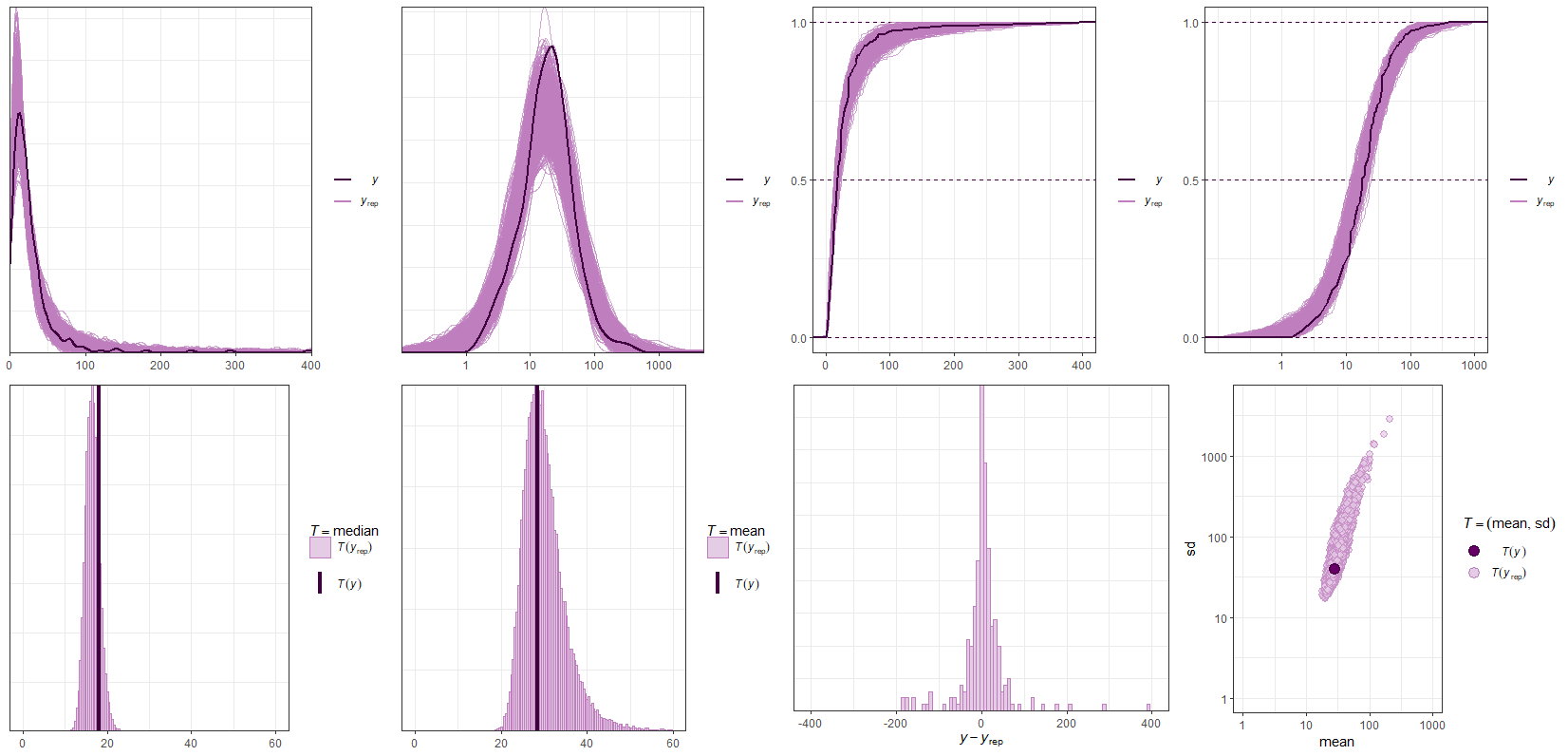


**Figure S4.** Posterior predictive checks performed using the full model for the joint datasets. The top row shows (from left to right) the kernel density estimates for 1000 datasets simulated on the normal and log scale, and the cumulative distribution function on the normal and log scale. The bottom row shows (from left to right) histograms of predicted median values (dark line representing the observed median), histograms of predicted mean values (dark line representing the observed mean), histogram of the residuals and scatterplots of predicted means and standard deviations on the log scale, each computed from 4000 draws from the posterior predictive distribution.


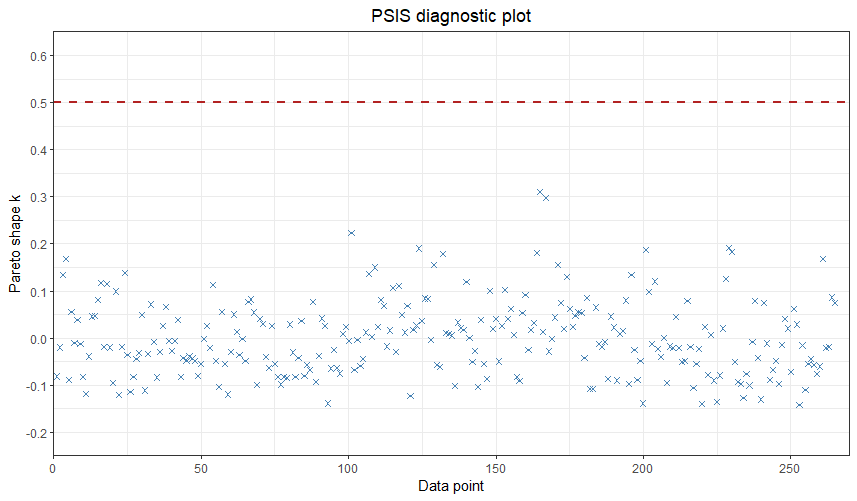


**Figure S5.** Pareto smoothed importance sampling plots for each data point. These values are used to assess the reliability of model estimates by highlighting which individual case detection delay data points are most influential on the posterior distribution. The plot shows that all data points were below the acceptable Pareto shape k threshold (0.5) for the diagnostic output of the model.


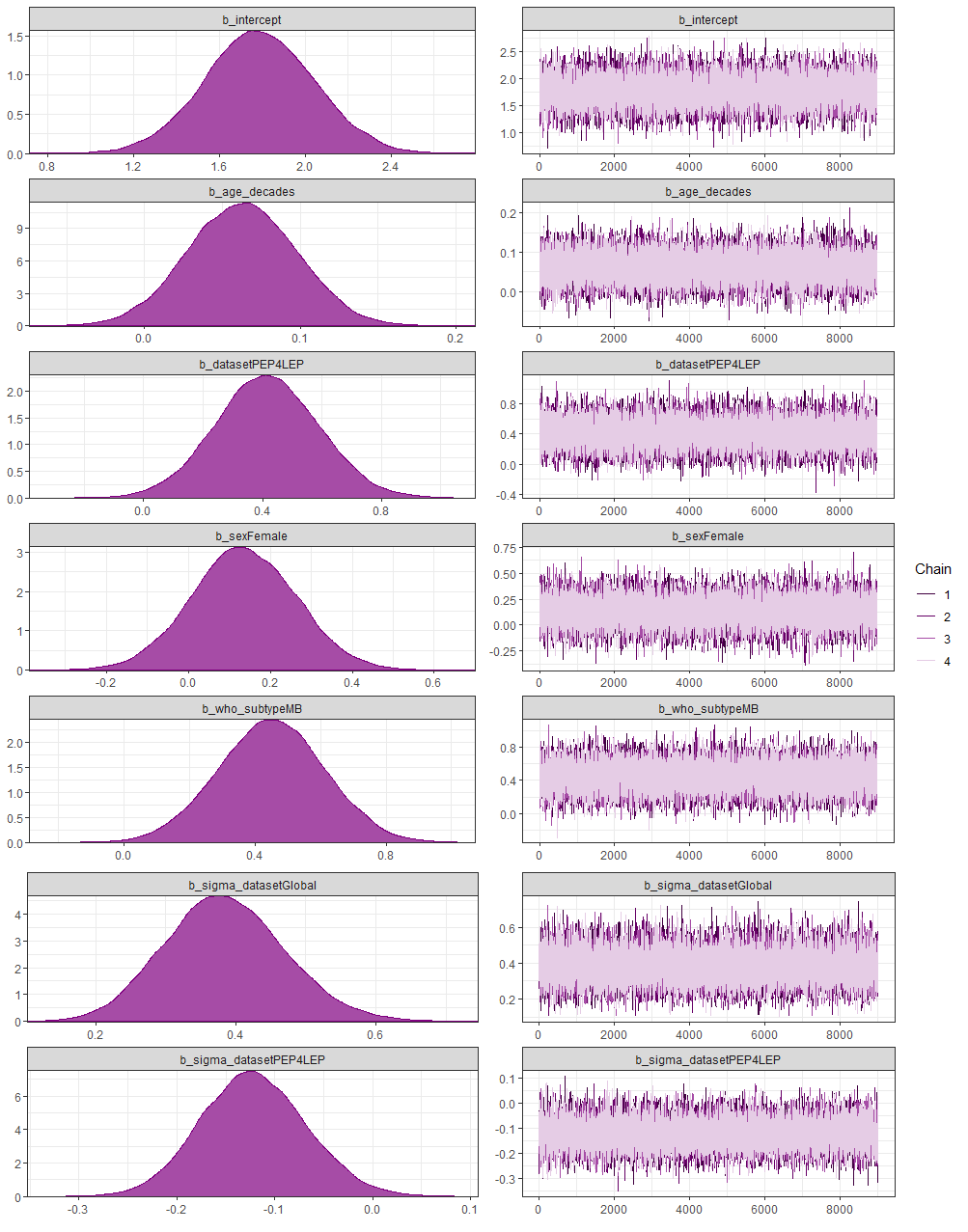


**Figure S6.** Density (left) and trace (right) plots for model parameters showing a stable sampling distribution across the four chains and convergence towards the posterior distribution. Model coefficients are expressed on the logarithmic scale.


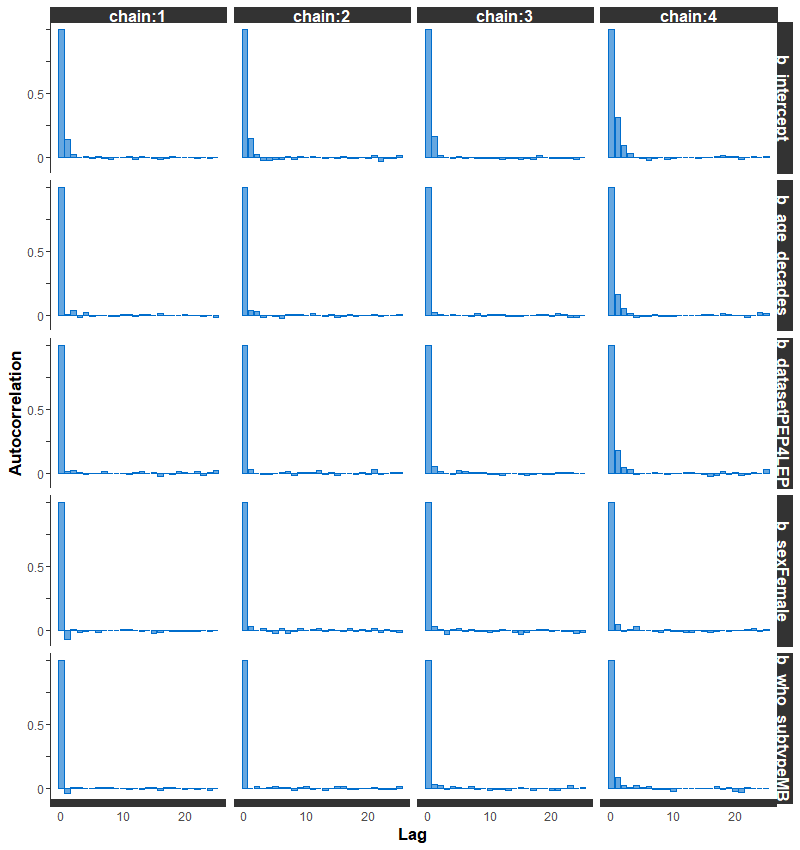


**Figure S7.** Autocorrelation plots for the model over 25 lags. The plot shows very low sample dependency for each covariate after only a few lags, with no need to sample from the posterior distribution for longer.
