# Supplementary material for: Establishing a standard method for analysing case detection delay in leprosy using a Bayesian modelling approach
Source: Infect Dis Poverty. 2023 Feb 20;12:12. doi: 10.1186/s40249-023-01065-4 (PMC9940321; doi:10.1186/s40249-023-01065-4)
Supplement: Supplementary file 2 — Additional file 2: Table S1. Details of the joint model run specifications in brms, including model formula and priors used for the intercept and covariate effects. [file 40249_2023_1065_MOESM2_ESM.docx]

**Additional file 2**

**Table S1.** Details of the joint model run specifications in brms, including model formula and priors used for the intercept and covariate effects.

| Model formula | |
| --- | --- |
| mod_brmslognorm_joint <- brm(bf(cdd \| cens(cen1) ~ 0 + intercept +  age_decades +  dataset +  sex +  who_subtype,  sigma ~ 0 + dataset),  iter = 10000,  warmup = 1000,  init = 0,  prior <- c(set_prior("normal(0,1)", class = "b")),  data = mydata_full,  family = "lognormal") | |
| Priors | |
| Covariate | **Prior** |
| intercept | Normal(0,1) |
| age_decades | Normal(0,1) |
| datasetPEP4LEP | Normal(0,1) |
| sexFemale | Normal(0,1) |
| who_subtypeMB | Normal(0,1) |
